# Supplementary material for: Structural basis for the assembly and energy transfer between the cyanobacterial PSI core and the double-layered IsiA proteins
Source: Nat Commun. 2025 Dec 20;17:592. doi: 10.1038/s41467-025-67295-2 (PMC12808642; doi:10.1038/s41467-025-67295-2)
Supplement: Supplementary file 1 — Supplementary Information [file 41467_2025_67295_MOESM1_ESM.pdf]

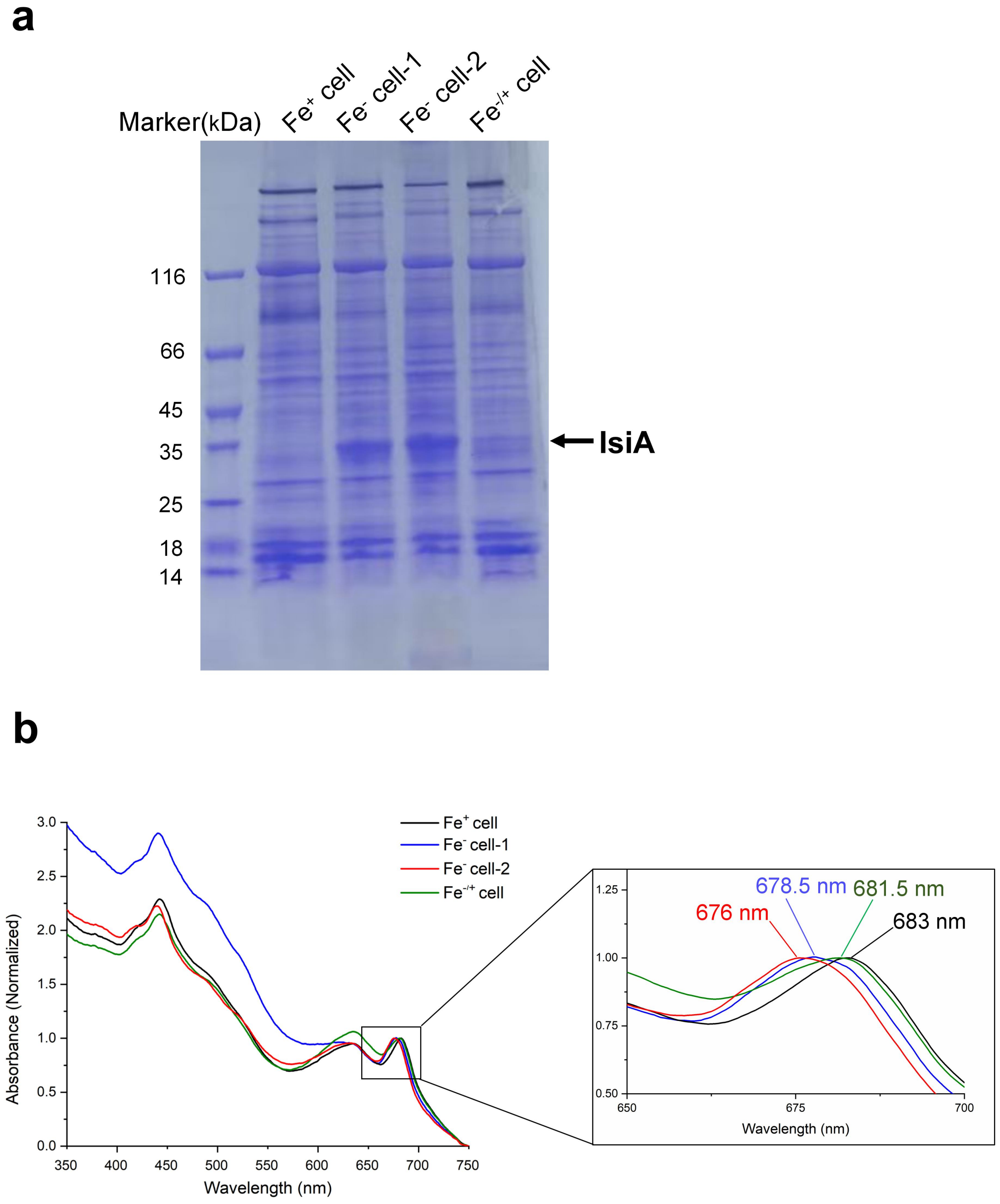

Supplementary Figure 1. Iron-dependent IsiA accumulation and reversibility of *T. elongatus* cells. (a) SDS-PAGE analysis of the cell extracts of different types of *T. elongatus* cells treated with various iron conditions. The Coomassie-stained band corresponding to IsiA indicated by an arrow was confirmed through mass spectrometry. (b) Room-temperature absorption spectra of cells grown under different iron conditions: Fe^- cell-1 (moderate limitation), Fe^- cell-2 (severe limitation), $Fe^{-/+}$ cell (iron restored after severe limitation), and Fe^+ cell (sufficiency). The Q_y region is shown enlarged.

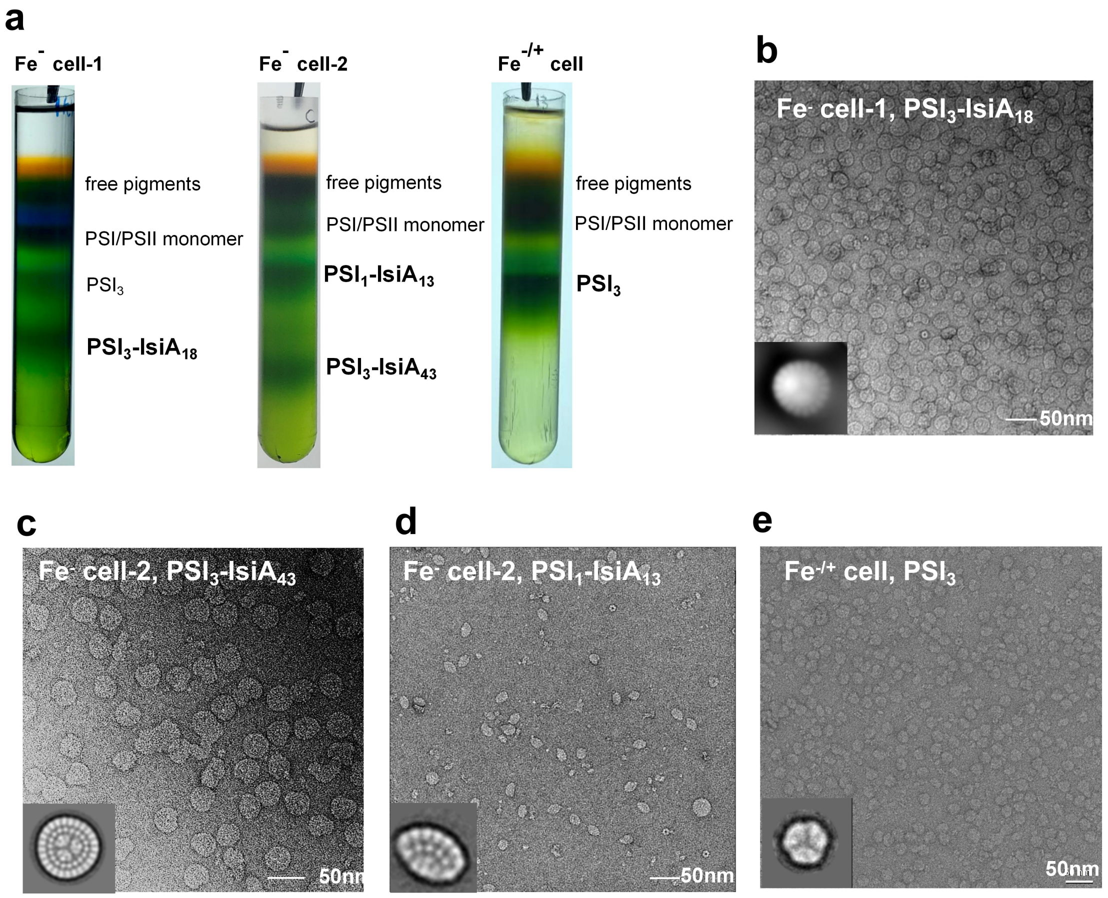

Supplementary Figure 2. Characterization of the isolated supercomplexes cultured under different iron concentrations. (a) Sucrose density gradient ultracentrifugation of thylakoid membranes isolated from T. *elongatus* cells grown under different iron conditions: Fe^- cell-1 (moderate limitation), Fe^- cell-2 (severe limitation), and $Fe^{-/+}$ cell (iron restored after severe limitation). Major bands are labeled and the PSI bands (bold labels) were collected for negative staining analysis. (b-e) Negative-staining electron microscopic images and 2D-classification results of PSI complexes obtained from Fe^- cell-1 (b), Fe^- cell-2 (c, d), and $Fe^{-/+}$ cell (e).

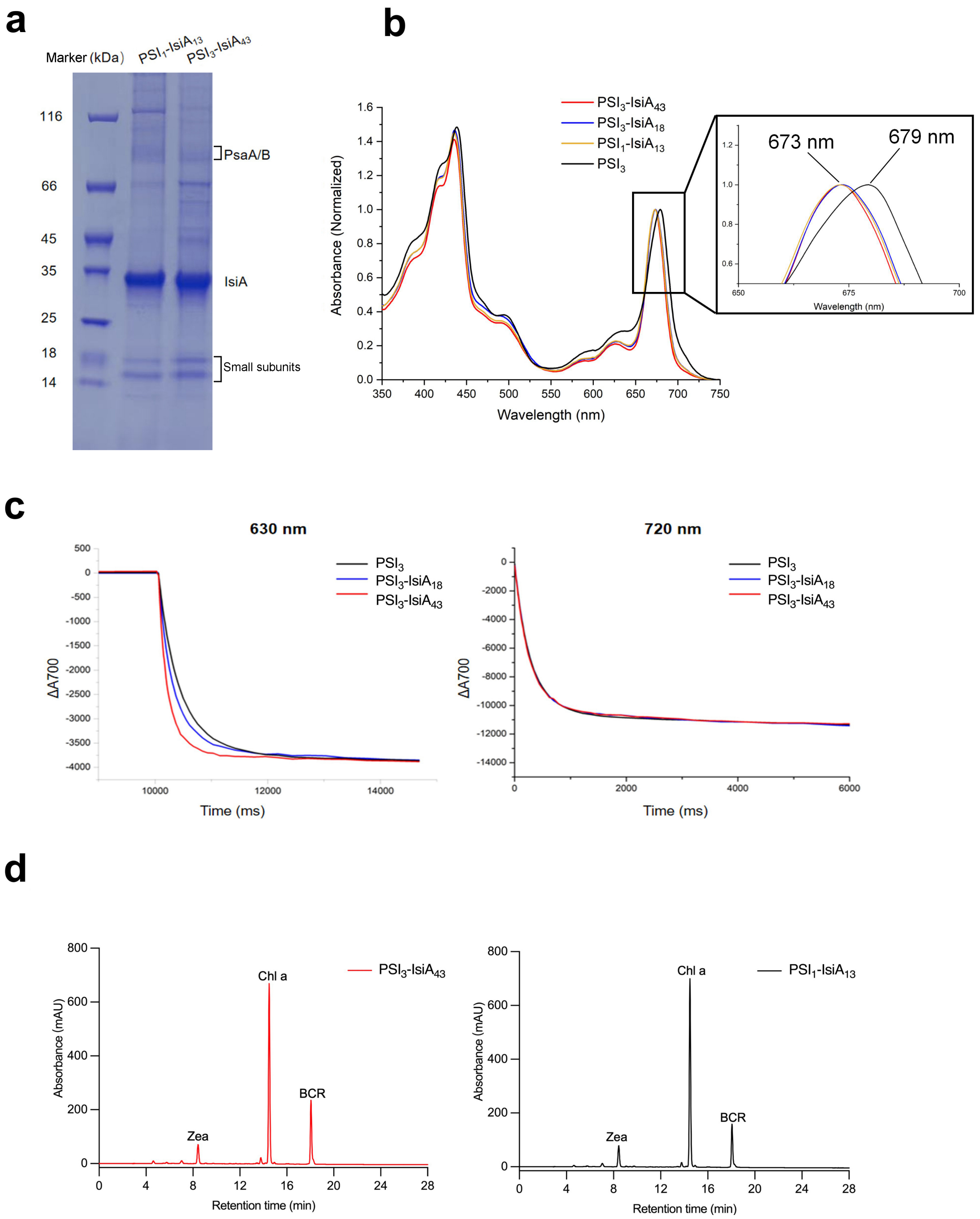

Supplementary Figure 3. Characterization of the PSI_3-$IsiA_{43}$ and PSI_1-$IsiA_{13}$ samples. (**a**) Gradient SDS-PAGE (4-20%) analysis of the PSI_3-$IsiA_{43}$ and PSI_1-$IsiA_{13}$ samples. (**b**) Comparison of the room-temperature absorption spectra of the PSI_3-$IsiA_{43}$, PSI_1-$IsiA_{13}$, PSI_3-$IsiA_{18}$, and PSI_3 samples, with the Qy absorption band magnified in the inset. (**c**) Fluorescence emission kinetics of the PSI_3-$IsiA_{43}$, PSI_3-$IsiA_{18}$ and PSI_3 samples measured by P700 oxidation at 630 nm and 720 nm. (**d**) Pigment analysis of the PSI_3-$IsiA_{43}$ and PSI_1-$IsiA_{13}$ samples by HPLC revealed three major peaks, identified as zeaxanthin (Zea), chlorophyll *a* (Chl *a*), and *β*-carotene (BCR).

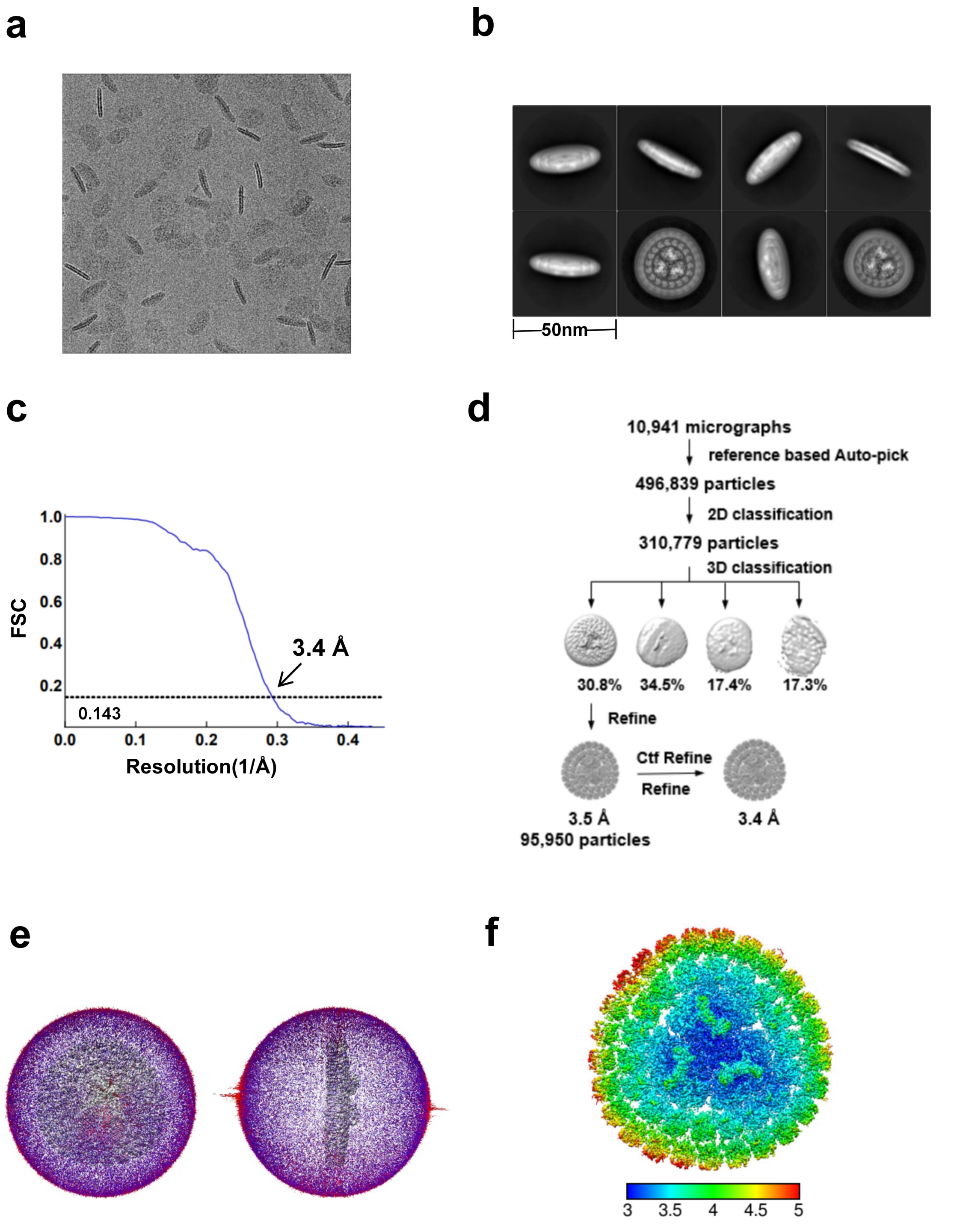

Supplementary Figure 4. Single-particle cryo-EM analysis of PSI_3-$IsiA_{43}$. (a) A representative cryo-EM micrograph of the PSI_3-$IsiA_{43}$ sample. Data were collected from four independently purified samples. (b) Representative reference-free 2D class averages. (c) Curve of Fourier shell correlation (FSC) against resolution. (d) Cryo-EM data processing workflow. (e) Euler angle distribution of particles included in the final refinement. (f) Local resolution map.

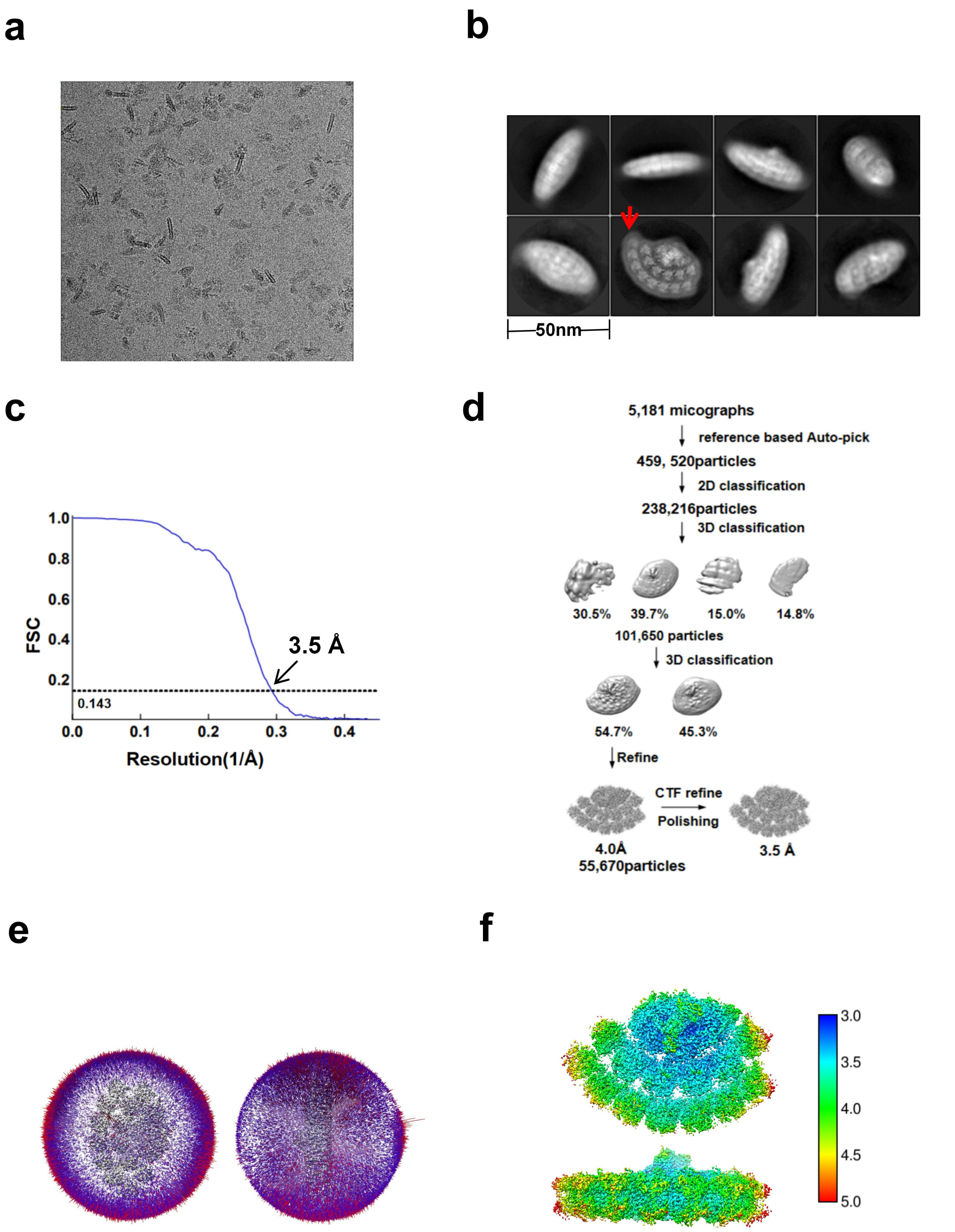

Supplementary Figure 5. Single-particle cryo-EM analysis of PSI$_1$-IsiA$_{13}$. (a) A representative cryo-EM micrograph of the PSI$_1$-IsiA$_{13}$ sample. Data were collected from two independently purified samples. (b) Representative reference-free 2D class averages. The red arrow indicates a density that potentially corresponds to IsiA-o-8, which has not been modeled in our PSI$_1$-IsiA$_{13}$ structure. (c) Curve of FSC against resolution. (d) Cryo-EM data processing workflow. (e) Euler angle distribution of particles included in the final refinement. (f) Local resolution map.

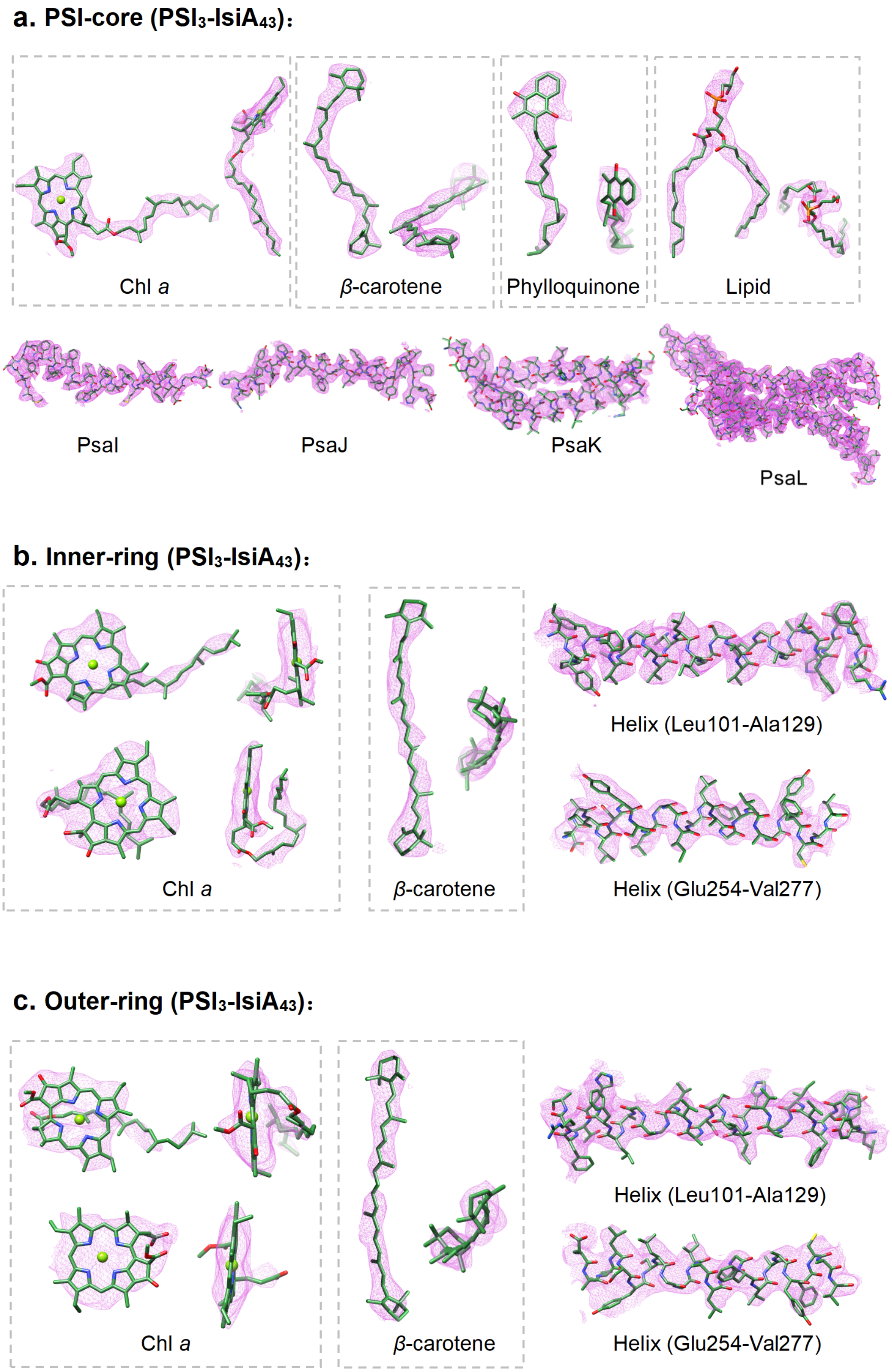
a. PSI-core (PSI3-IsiA43):
Chl a
β-carotene
Phylloquinone
Lipid
PsaI
PsaJ
PsaK
PsaL
b. Inner-ring (PSI3-IsiA43):
Helix (Leu101-Ala129)
Chl a
β-carotene
Helix (Glu254-Val277)
c. Outer-ring (PSI3-IsiA43):
Helix (Leu101-Ala129)
Chl a
β-carotene
Helix (Glu254-Val277)

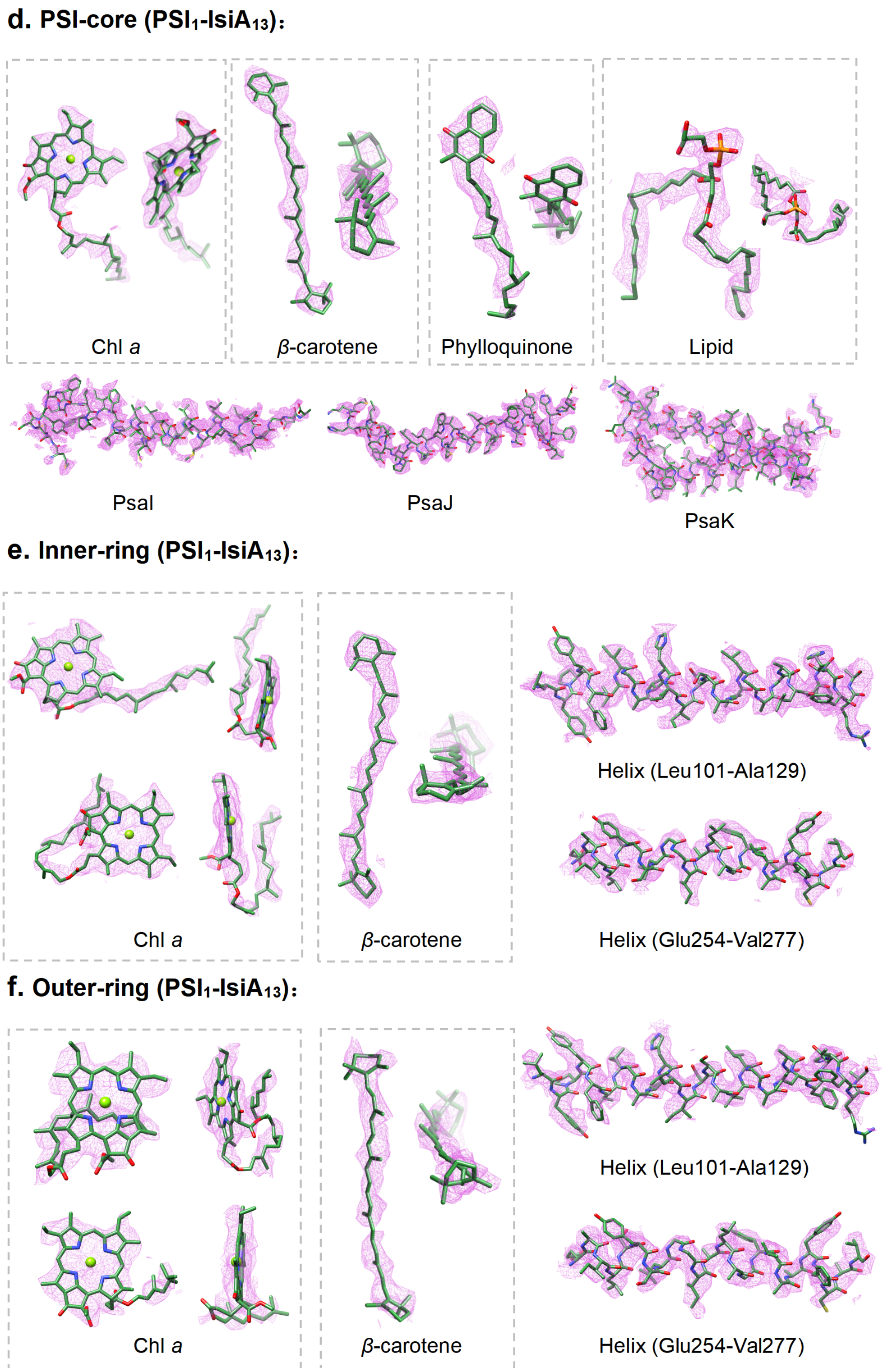

Supplementary Figure 6. Representative Cryo-EM densities. (a-c) Selected regions showing representative peptide fragments and cofactors from the core (a), the inner IsiA (b) and the outer IsiA (c) from the PSI_3-$IsiA_{43}$ structure. (d-f) Selected regions showing representative peptide fragments and cofactors from the core (d), the inner IsiA (e) and the outer IsiA (f) from the PSI_1-$IsiA_{13}$ structure. Ligands are shown in two different views.

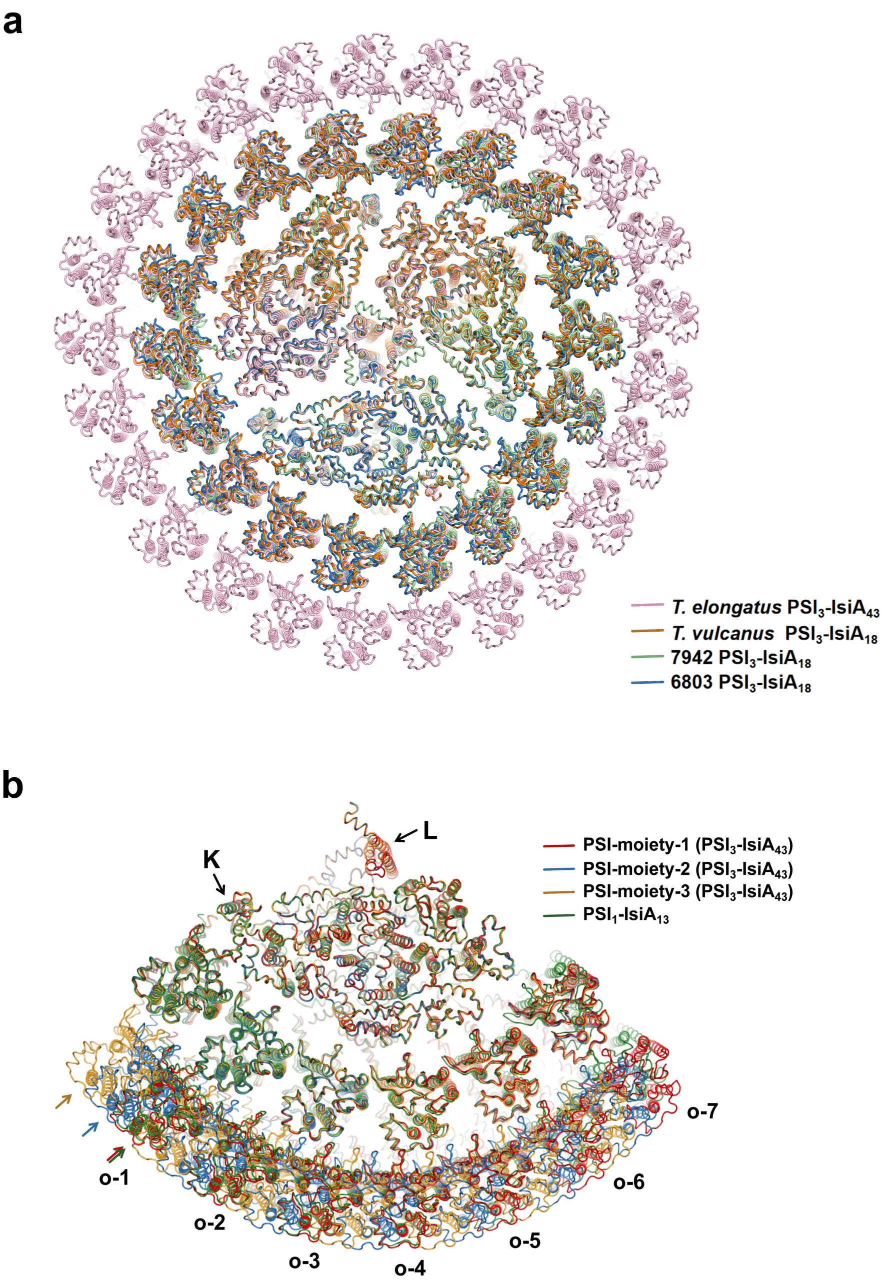

Supplementary Figure 7. Structural comparison of PSI-IsiA complexes. (a) Structural comparison of the PSI$_3$-IsiA$_{18}$ parts of PSI-IsiA complexes from different cyanobacterial species. The PSI$_3$-IsiA$_{43}$ complex (pink), the PSI$_3$-IsiA$_{18}$ complexes from *T. vulcanus* (orange), *Synechococcus elongatus* PCC 7942 (7942, green), and *Synechocystis* sp. PCC 6803 (6803, blue) were aligned by one PsaA subunit. (b) Comparison of three PSI-moieties of PSI$_3$-IsiA$_{43}$ (red, blue and orange) and PSI$_1$-IsiA$_{13}$ complex (deep green). Colored arrows indicate the position of the same transmembrane helix of the IsiA subunit corresponding to IsiA-o-1 across different structures.

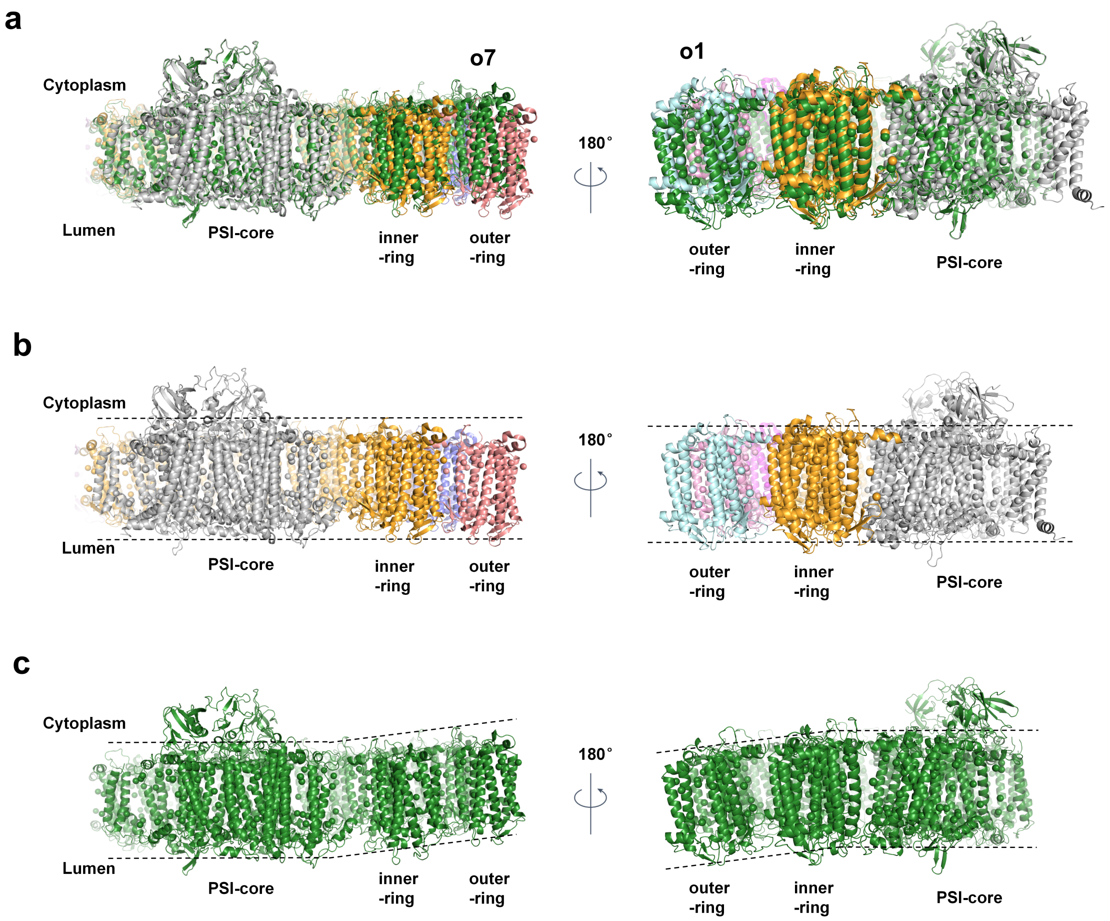

Supplementary Figure 8. Membrane curvature of PSI$_1$-IsiA$_{13}$ versus PSI$_3$-IsiA$_{43}$. (a) Comparison of PSI$_1$-IsiA$_{13}$ (shown in deep green) and PSI$_3$-IsiA$_{43}$ (colored as in Fig. 1b), viewed from the membrane plane. The o7 end (left) and the o1 end (right) of PSI$_1$-IsiA$_{13}$ shift vertically in opposite directions relative to the corresponding subunits in PSI$_3$-IsiA$_{43}$. (b, c) Side views of PSI$_3$-IsiA$_{43}$ (b) and PSI$_1$-IsiA$_{13}$ (c), viewed along the same orientation as in (a). Black dashed lines indicate the putative membrane plane.

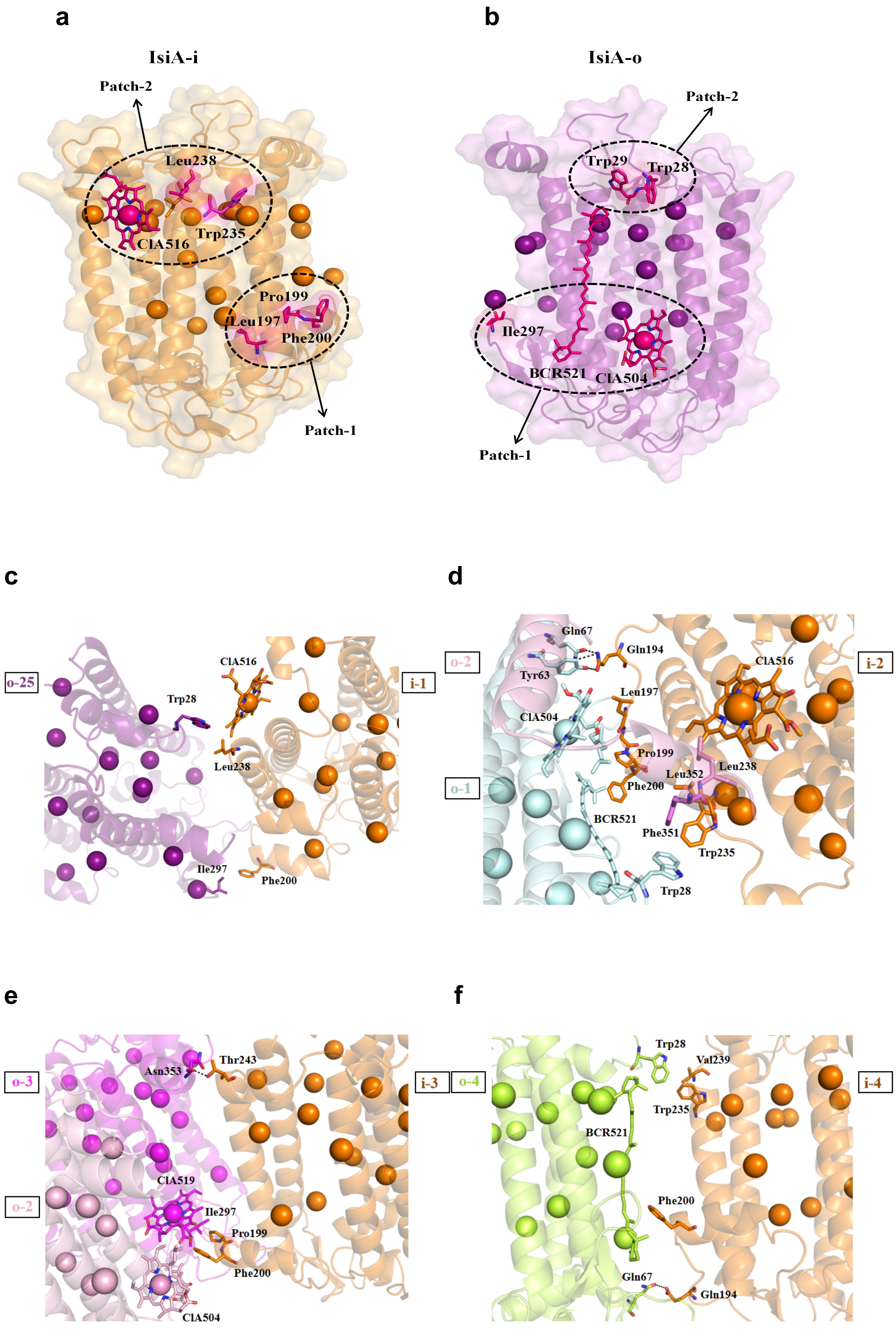
a
IsiA-i
Patch-2
Leu238
ClA516
Trp235
Pro199
Leu197
Phe200
Patch-1
b
IsiA-o
Patch-2
Trp29
Trp28
Ile297
BCR521
ClA504
Patch-1
c
o-25
i-1
ClA516
Trp28
Leu238
Ile297
Phe200
d
o-2
i-2
o-1
Gln67
Gln194
Tyr63
Leu197
ClA516
ClA504
Pro199
Leu352
Leu238
Phe200
BCR521
Phe351
Trp235
Trp28
e
o-3
i-3
o-2
Asn353
Thr243
ClA519
Ile297
Pro199
Phe200
ClA504
f
o-4
i-4
Trp28
Val239
Trp235
BCR521
Phe200
Gln67
Gln194

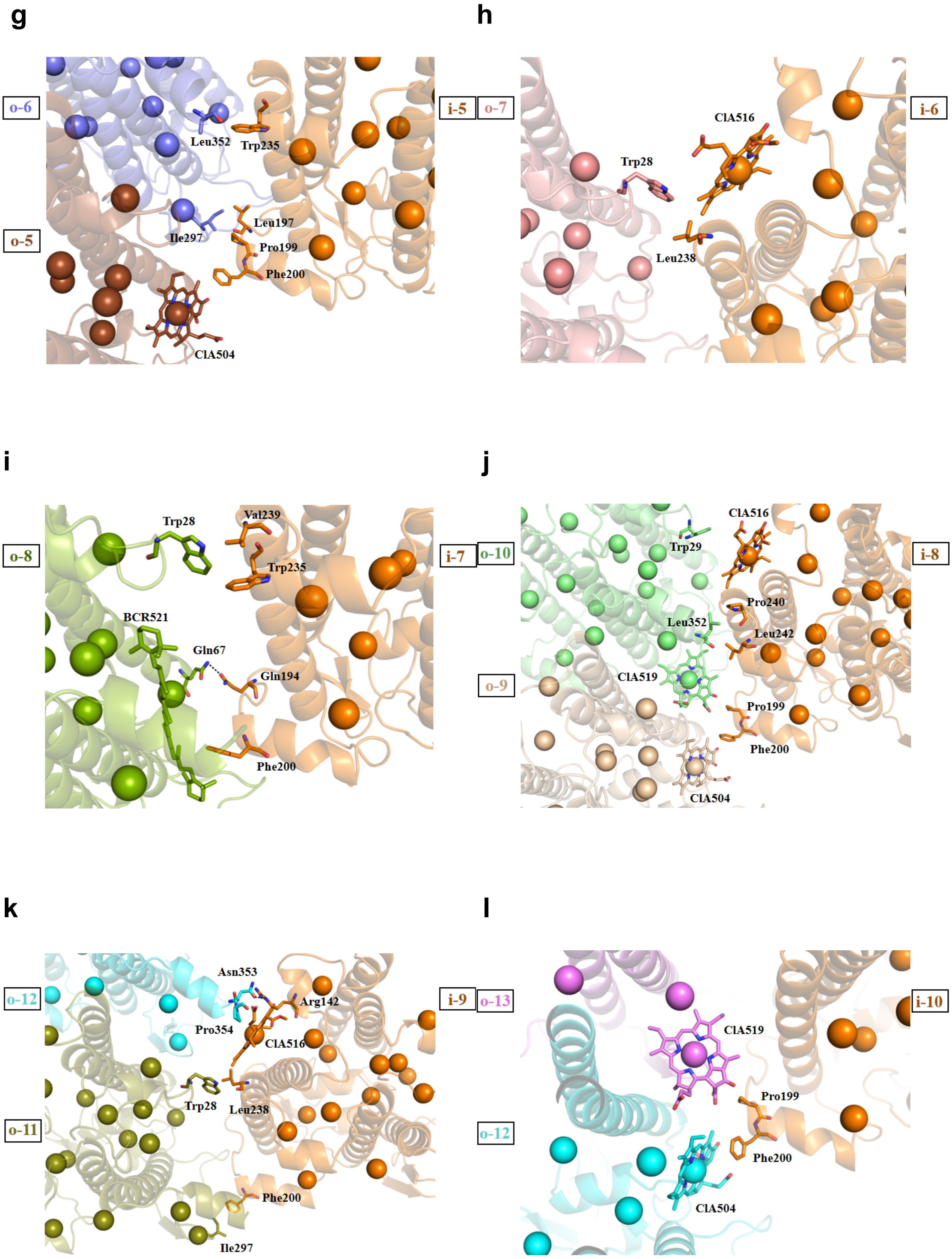
g
o-6
Leu352
Trp235
i-5
o-5
Ile297
Leu197
Pro199
Phe200
ClA504
h
o-7
ClA516
i-6
Trp28
Leu238
i
o-8
Trp28
Val239
Trp235
BCR521
Gln67
Gln194
Phe200
j
i-7
o-10
ClA516
Trp29
i-8
Pro240
Leu352
Leu242
o-9
ClA519
Pro199
Phe200
ClA504
k
o-12
Asn353
Arg142
Pro354
ClA516
i-9
o-11
Trp28
Leu238
Phe200
Ile297
l
o-13
i-10
ClA519
Pro199
o-12
Phe200
ClA504

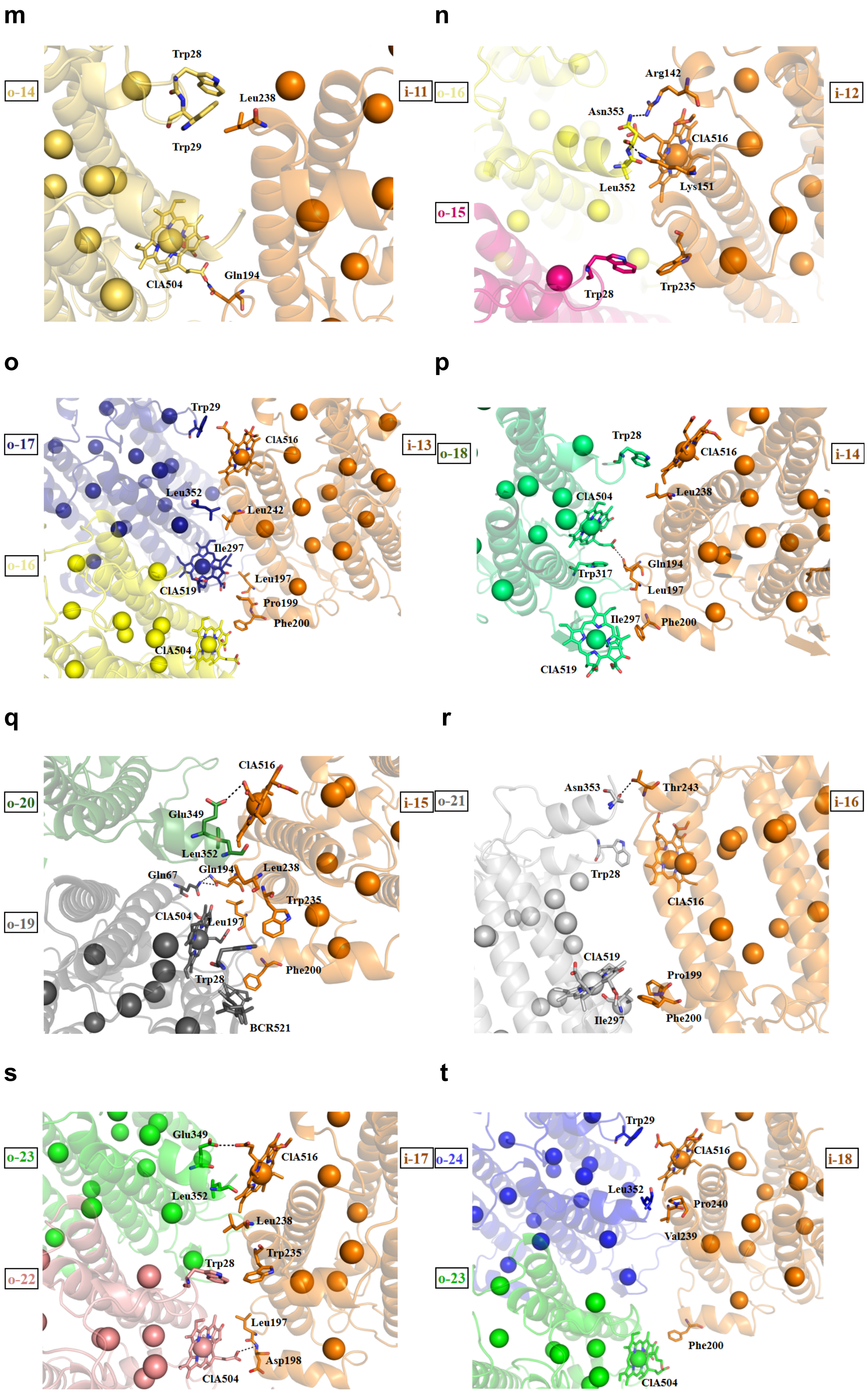
m
o-14
Trp28
Leu238
Trp29
ClA504
Gln194
i-11
n
o-16
Arg142
Asn353
ClA516
Leu352
Lys151
i-12
o-15
Trp28
Trp235
o
o-17
Trp29
ClA516
Leu352
Leu242
Ile297
o-16
ClA519
Leu197
Pro199
Phe200
ClA504
i-13
p
o-18
Trp28
ClA516
Leu238
ClA504
Gln194
Trp317
Leu197
Ile297
Phe200
ClA519
i-14
q
o-20
ClA516
Glu349
Leu352
Gln194
Leu238
Gln67
o-19
Trp235
ClA504
Leu197
Trp28
Phe200
BCR521
i-15
r
o-21
Asn353
Thr243
Trp28
ClA516
ClA519
Pro199
Ile297
Phe200
i-16
s
o-23
Glu349
ClA516
Leu352
Leu238
Trp235
Trp28
o-22
Leu197
Asp198
ClA504
i-17
t
o-24
Trp29
ClA516
Leu352
Pro240
Val239
o-23
Phe200
ClA504
i-18

Supplementary Figure 9. The hydrophobic and hydrogen-bond interactions between the inner and outer IsiA subunits in PSI_3-$IsiA_{43}$. (a, b) Two common patches (patch-1 and patch-2) involved in the inter-ring interactions, shown in the inner-IsiA subunits (a) and in the outer-IsiA subunits (b). The patches are highlighted with black dashed circles. The amino acids and pigments involved in the interactions are shown as sticks, and other Chl molecules are shown as spheres at their central Mg positions. (c-t) The detailed interactions of each inner-outer IsiA pair. The inner IsiA subunits are colored in orange. The IsiA subunits in the outer ring are in the same color codes as in Fig. 1b. The hydrogen-bonds are shown in black dashed lines.

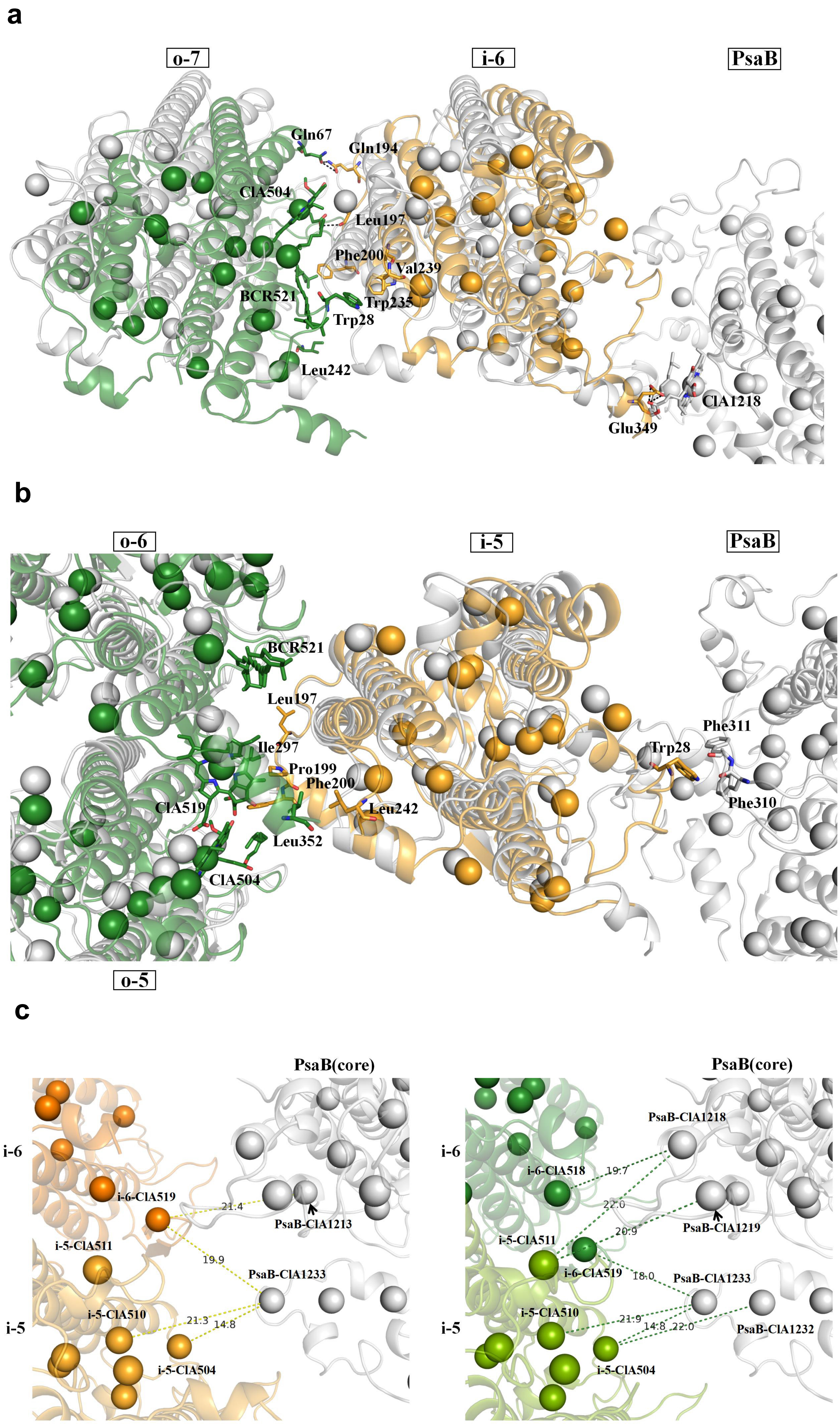
a
o-7
i-6
PsaB
Gln67
Gln194
ClA504
Leu197
Phe200
Val239
BCR521
Trp235
Trp28
Leu242
ClA1218
Glu349
b
o-6
i-5
PsaB
BCR521
Leu197
Ile297
Pro199
Phe200
ClA519
Leu242
Leu352
ClA504
Phe311
Trp28
Phe310
o-5
c
PsaB(core)
i-6
i-6-ClA519
21.4
PsaB-ClA1213
i-5-ClA511
19.9
PsaB-ClA1233
i-5
i-5-ClA510
21.3
14.8
i-5-ClA504
PsaB(core)
PsaB-ClA1218
i-6
i-6-ClA518
19.7
22.0
PsaB-ClA1219
20.9
i-5-ClA511
i-6-ClA519
18.0
PsaB-ClA1233
i-5
i-5-ClA510
21.9
14.8
22.0
PsaB-ClA1232
i-5-ClA504

Supplementary Figure 10. Comparison of the structure and subunit interactions between PSI_1-$IsiA_{13}$ and PSI_3-$IsiA_{43}$. (a, b) Structural comparison of PSI_3-$IsiA_{43}$ complex (gray) and PSI_1-$IsiA_{13}$ complex (green for the outer ring and orange for the inner ring), aligned using the PsaA subunits. Panel (a) shows the region involving IsiA-i-6, IsiA-o-7, and PsaB, whereas panel (b) shows IsiA-i-5, IsiA-o-5, IsiA-o-6, and PsaB. The residues involved in the interactions are shown as sticks, and Chl molecules are represented as spheres at their central Mg positions. (c) The closely associated interfacial Chl molecules between IsiA-i-5~6 and PsaB in PSI_3-$IsiA_{43}$ (left) and PSI_1-$IsiA_{13}$ (right) are linked by dashed lines (Mg-Mg distance≤ 22 Å), with the Mg-Mg distances labeled (in Å).

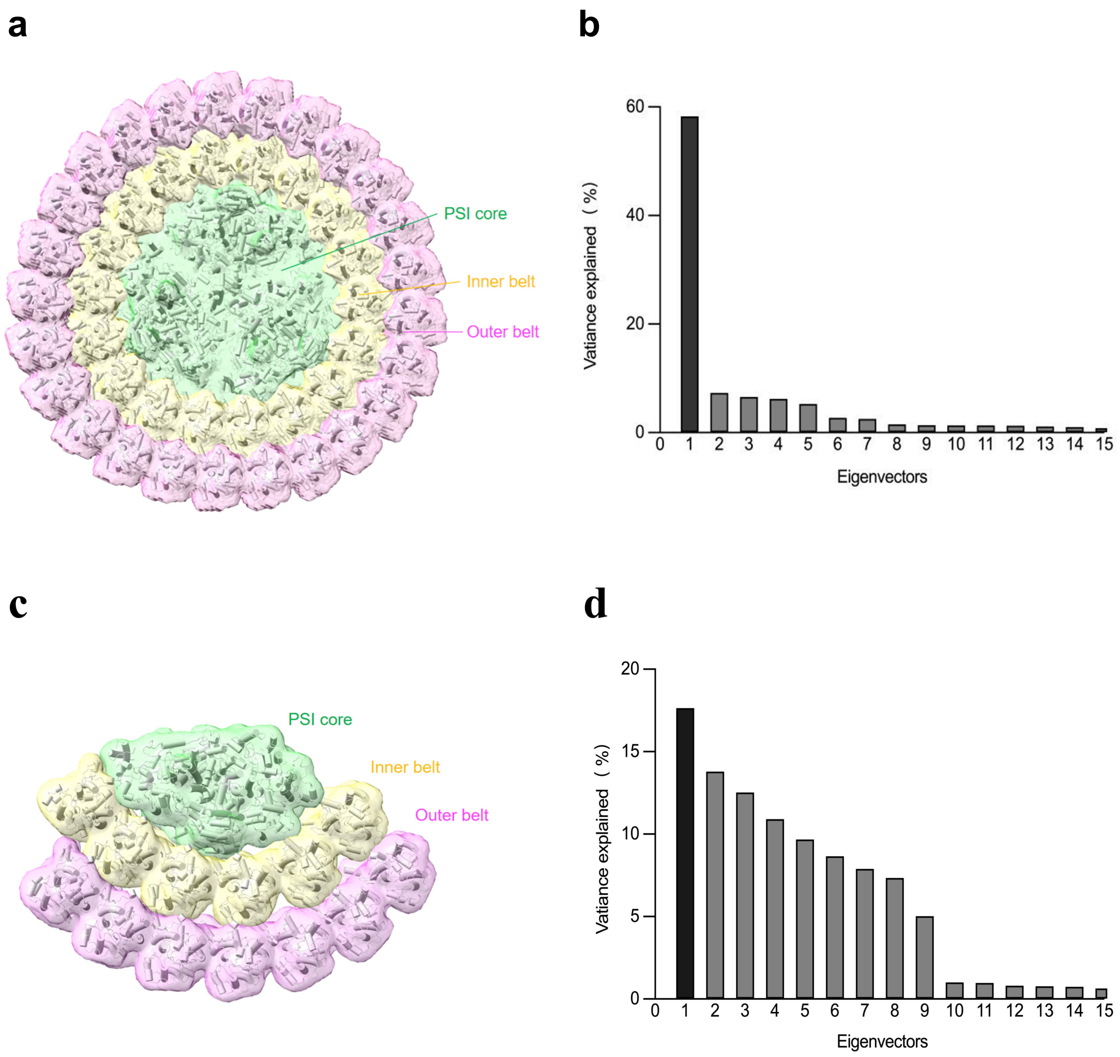

Supplementary Figure 11. Multibody refinement results of PSI$_3$-IsiA$_{43}$ and PSI$_1$-IsiA$_{13}$. (a) Three bodies used for multibody refinement in PSI$_3$-IsiA$_{43}$: PSI core, inner belt and outer belt. (b) The contributions of all 15 eigenvectors to the variance in PSI$_3$-IsiA$_{43}$. (c) Three bodies used for multibody refinement in PSI$_1$-IsiA$_{13}$: PSI-core, inner belt and outer belt. (d) The contributions of all 15 eigenvectors to the variance in PSI$_1$-IsiA$_{13}$.

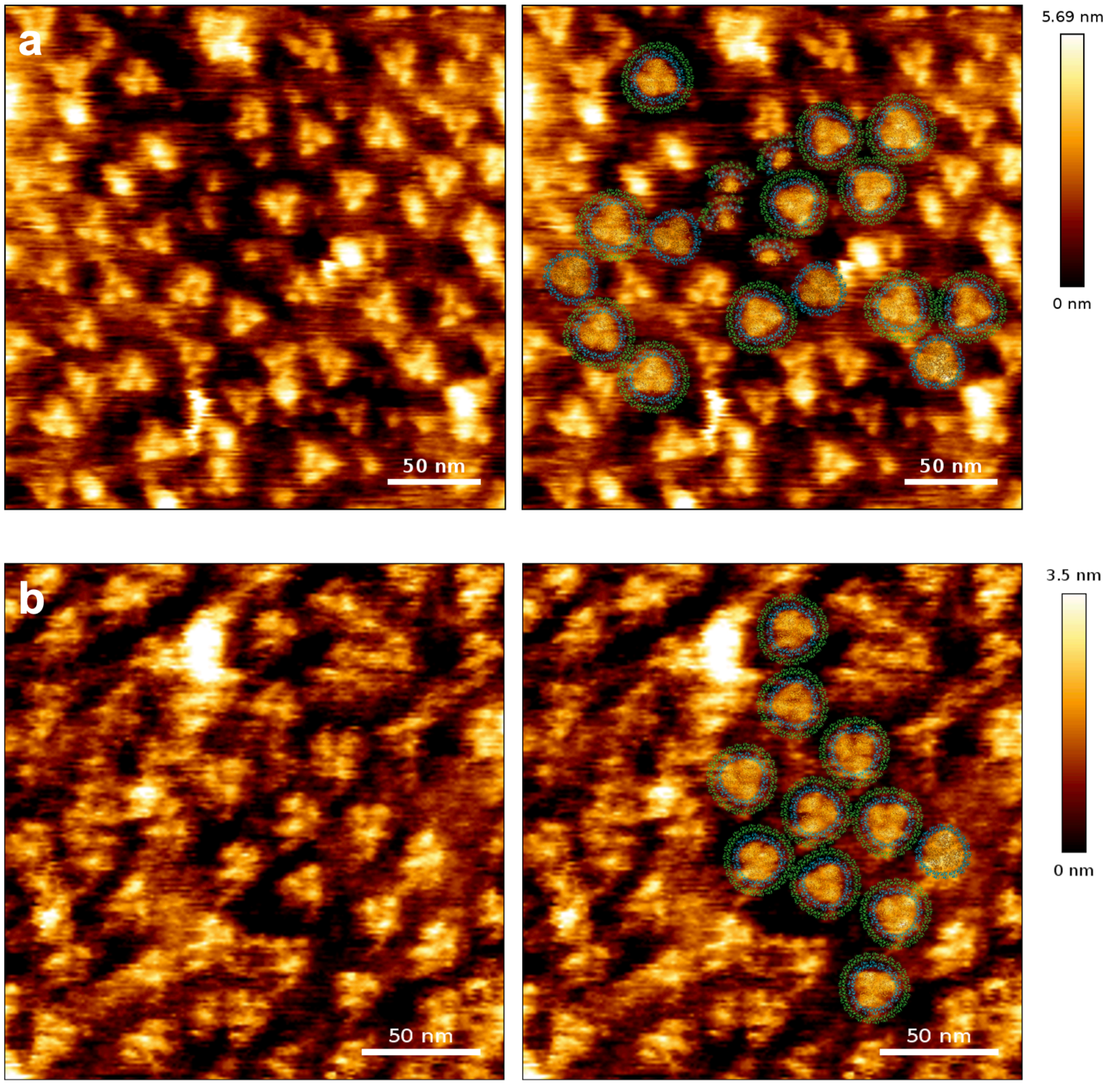

Supplementary Figure 12. AFM topographs of native thylakoid membranes isolated from the iron-starved *T. elongatus* cells (Fe^- cell-2) revealing the arrangement of PSI-IsiA supercomplexes. (a, b) Two different membrane patches. Right panels: PSI-IsiA complexes with double IsiA rings/layers and single IsiA rings are annotated by superimposing the PSI_3-$IsiA_{43}$, PSI_3-$IsiA_{18}$ (PSI_3-$IsiA_{43}$ with the outer IsiA ring removed), and PSI_1-$IsiA_{13}$ structures on the AFM image.

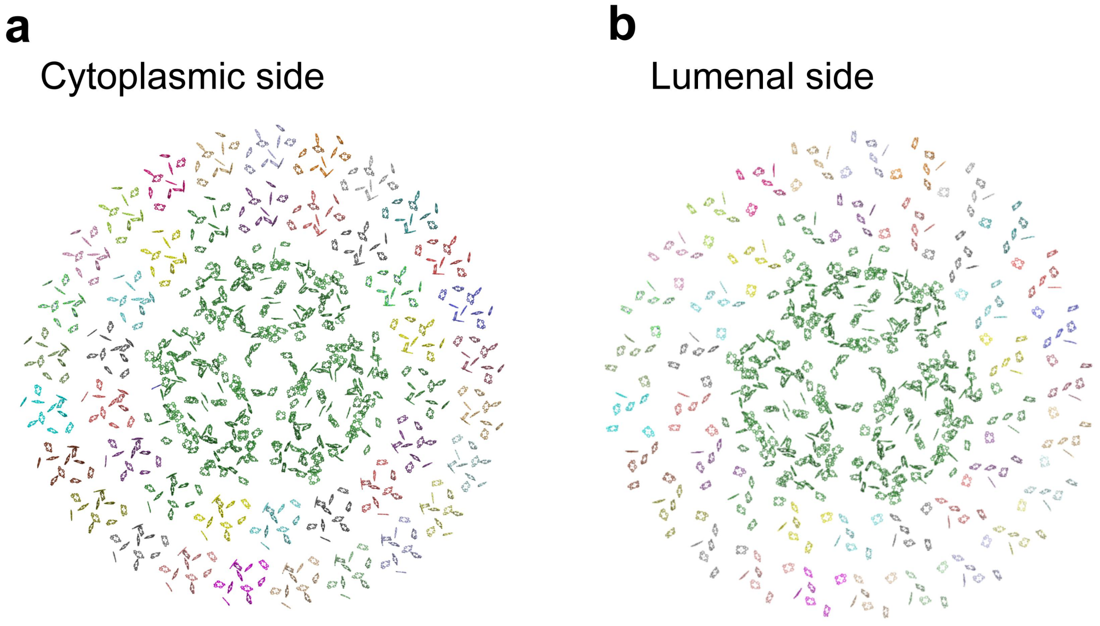

Supplementary Figure 13. Distribution of Chl molecules in PSI_3-$IsiA_{43}$. It is viewed from the cytoplasmic side (a) and the lumenal side (b).

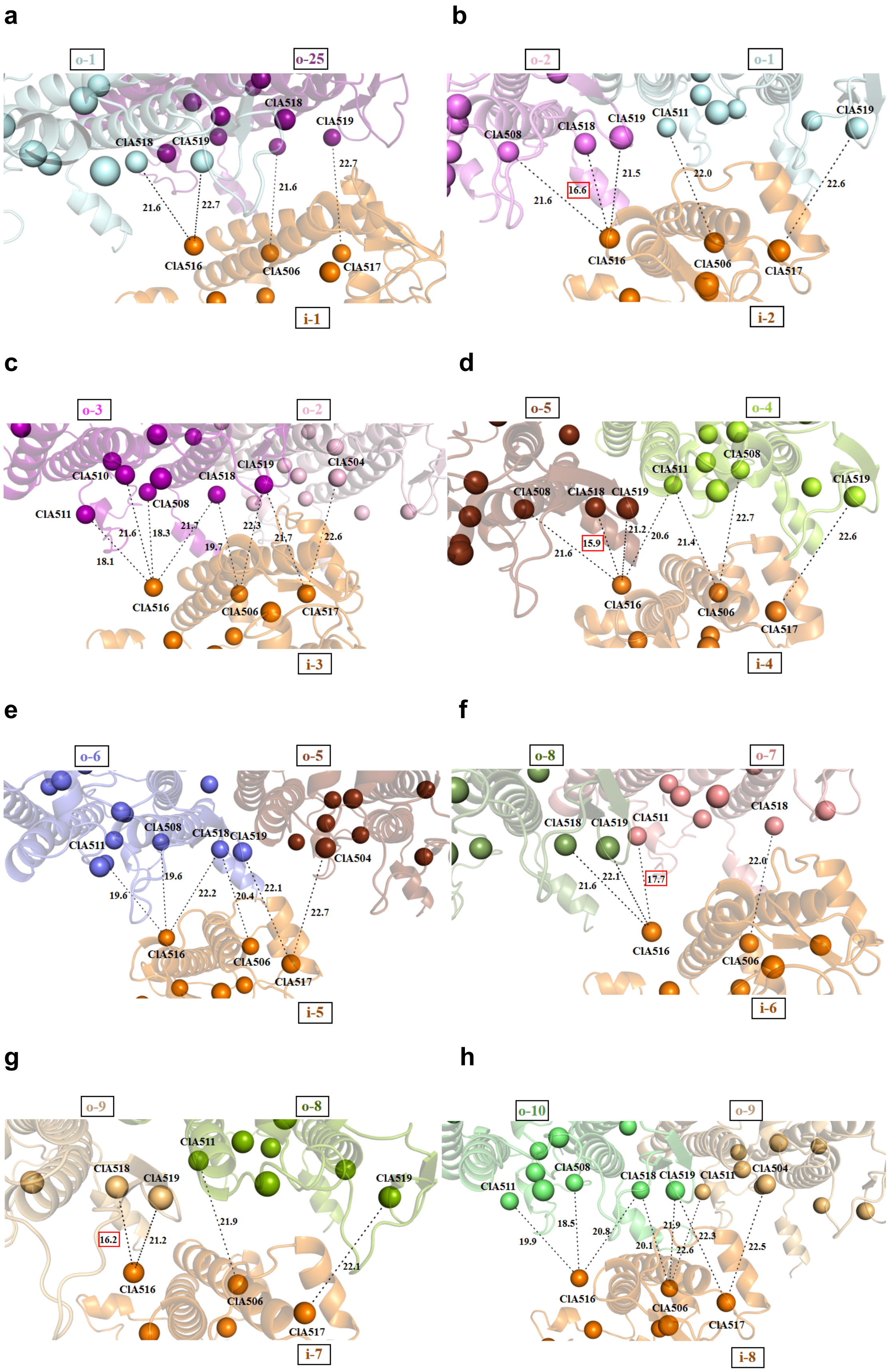
a
o-1
o-25
ClA518
ClA519
ClA518
ClA519
22.7
21.6
21.6
22.7
ClA516
ClA506
ClA517
i-1
b
o-2
o-1
ClA508
ClA518
ClA519
ClA511
ClA519
16.6
21.5
22.0
22.6
21.6
ClA516
ClA506
ClA517
i-2
c
o-3
o-2
ClA510
ClA518
ClA519
ClA504
ClA508
ClA511
21.6
18.3
21.7
22.3
21.7
22.6
19.7
18.1
ClA516
ClA506
ClA517
i-3
d
o-5
o-4
ClA508
ClA511
ClA508
ClA518
ClA519
ClA519
15.9
21.2
20.6
21.4
22.7
22.6
21.6
ClA516
ClA506
ClA517
i-4
e
o-6
o-5
ClA508
ClA518
ClA519
ClA511
ClA504
19.6
19.6
22.2
20.4
22.1
22.7
ClA516
ClA506
ClA517
i-5
f
o-8
o-7
ClA518
ClA519
ClA511
ClA518
22.0
21.6
22.1
17.7
ClA516
ClA506
i-6
g
o-9
o-8
ClA511
ClA518
ClA519
ClA519
21.9
16.2
21.2
22.1
ClA516
ClA506
ClA517
i-7
h
o-10
o-9
ClA508
ClA518
ClA519
ClA511
ClA504
ClA511
18.5
20.8
21.9
22.3
22.5
19.9
20.1
22.6
ClA516
ClA506
ClA517
i-8

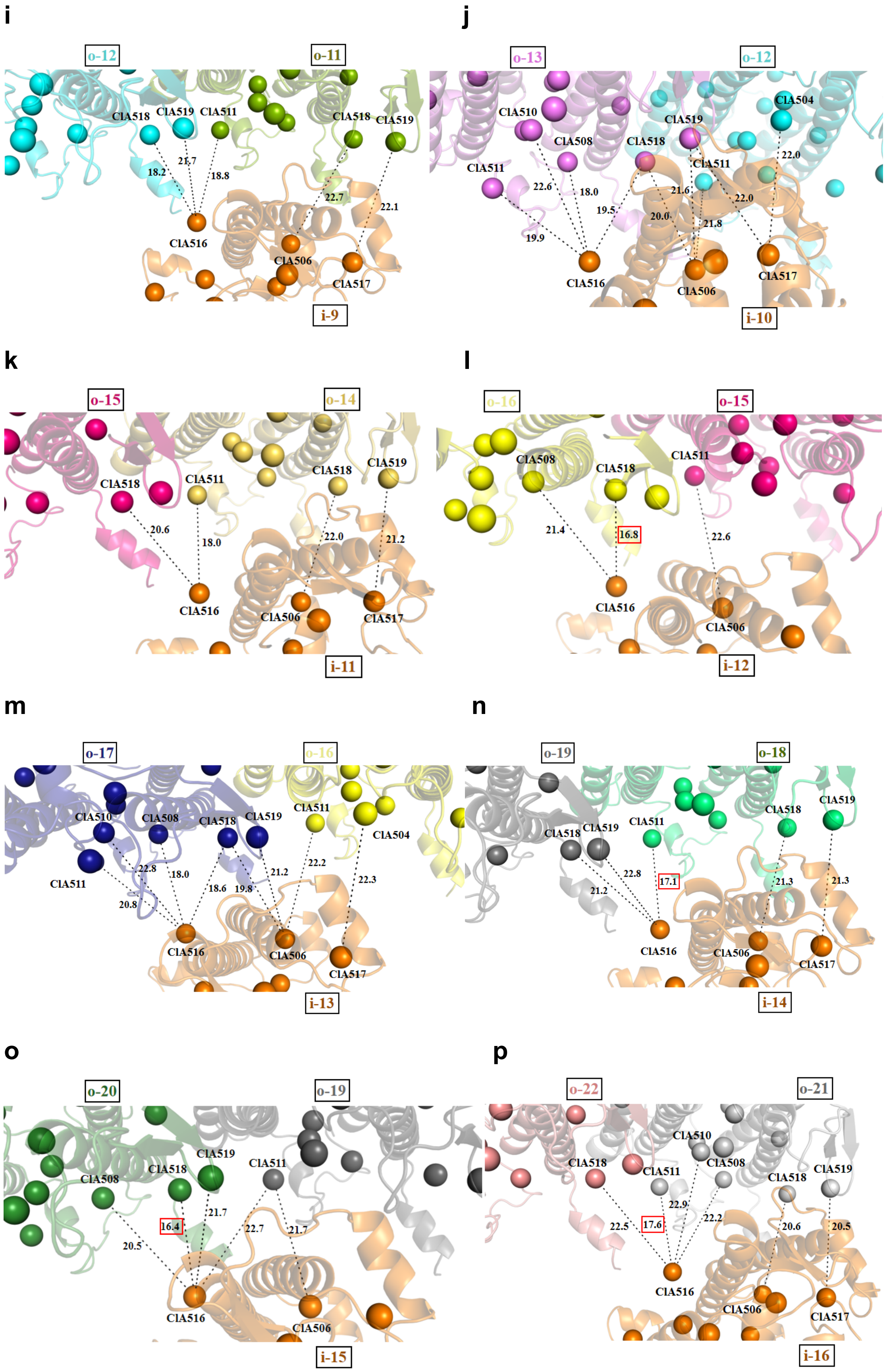
i
o-12
o-11
ClA518
ClA519
ClA511
ClA518
ClA519
18.2
21.7
18.8
22.7
22.1
ClA516
ClA506
ClA517
i-9
j
o-13
o-12
ClA504
ClA510
ClA508
ClA518
ClA519
ClA511
ClA511
22.0
22.6
18.0
21.6
22.0
19.5
20.0
21.8
19.9
ClA516
ClA506
ClA517
i-10
k
o-15
o-14
ClA518
ClA519
ClA518
ClA511
20.6
18.0
22.0
21.2
ClA516
ClA506
ClA517
i-11
l
o-16
o-15
ClA508
ClA518
ClA511
21.4
16.8
22.6
ClA516
ClA506
i-12
m
o-17
o-16
ClA510
ClA508
ClA518
ClA519
ClA511
ClA504
ClA511
22.8
18.0
21.2
22.2
22.3
18.6
19.8
20.8
ClA516
ClA506
ClA517
i-13
n
o-19
o-18
ClA519
ClA518
ClA519
ClA511
ClA518
22.8
17.1
21.3
21.3
21.2
ClA516
ClA506
ClA517
i-14
o
o-20
o-19
ClA519
ClA518
ClA511
ClA508
16.4
21.7
22.7
21.7
20.5
ClA516
ClA506
i-15
p
o-22
o-21
ClA510
ClA518
ClA511
ClA508
ClA519
ClA518
22.9
22.5
17.6
22.2
20.6
20.5
ClA516
ClA506
ClA517
i-16

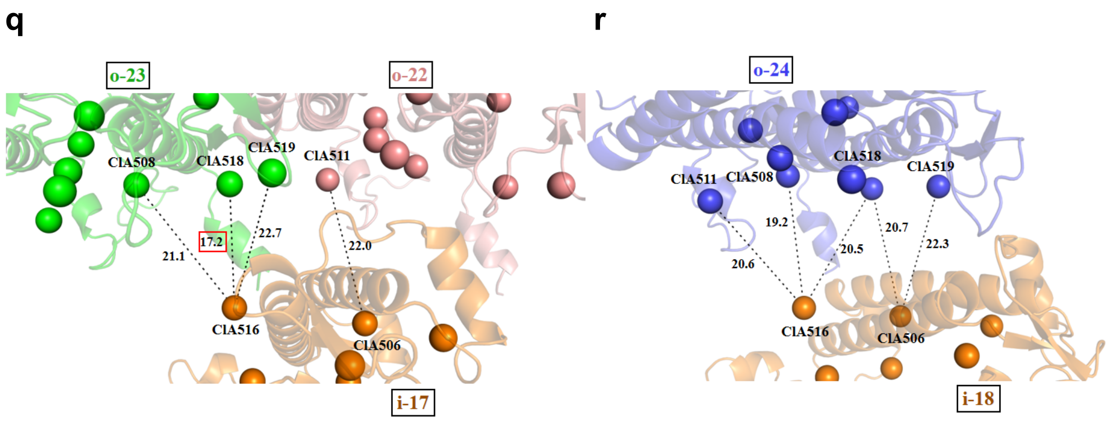

Supplementary Figure 14. Potential EET pathways between the inner and outer IsiA subunits. Interfacial Chl pairs (Mg-Mg distance ⩽ 23 Å) are connected by black dashed lines, with the distances labeled in Å. Distances < 18 Å are highlighted by red boxes. Inner IsiA subunits are shown in orange, while outer-ring IsiA subunits follow the same color scheme as in Fig. 1b.

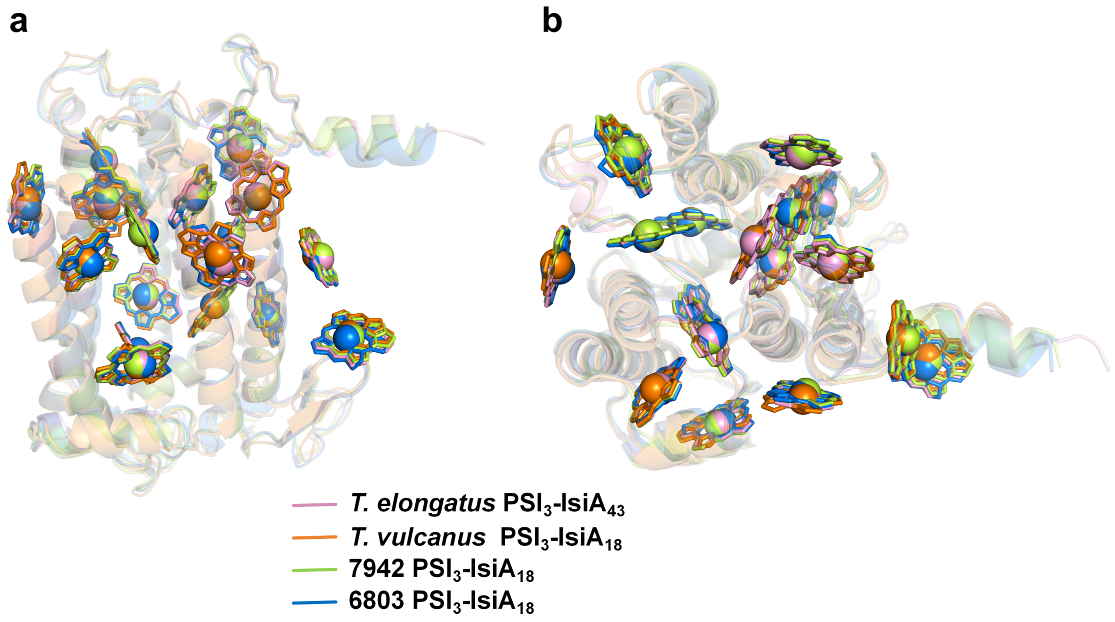

Supplementary Figure 15. Structural comparison of the IsiA proteins across different PSI-IsiA complexes. (a, b) Side view (a) and Top view (b) of IsiA subunits from PSI-IsiA complexes of different cyanobacterial species. IsiA subunits in the PSI_3-$IsiA_{43}$ complex from *T. elongatus* (this study, pink) and the PSI_3-$IsiA_{18}$ complexes from *T. vulcanus* (PDB code 6K33 [https://doi.org/10.2210/pdb6K33/pdb], orange), *Synechococcus elongatus* PCC 7942 (PDB code 6KIG [https://doi.org/10.2210/pdb6KIG/pdb], green), and *Synechocystis* sp. PCC 6803 (PDB code 7UMH [https://doi.org/10.2210/pdb7UMH/pdb], blue) were aligned.

Supplementary Table 1. Cryo-EM data collection, refinement and validation statistics.

	**PSI_3-$IsiA_{43}$** (EMDB-63527) (PDB 9LZJ)	**PSI_1-$IsiA_{13}$** (EMDB-63528) (PDB 9LZK)
**Data collection and processing**		
Magnification	130,000	130,000
Voltage (kV)	300	200
Electron exposure ($e^-/Å^2$)	60	60
Defocus range (μm)	1.5-2.5	1.2-2.5
Pixel size (Å)	1.04	1
Symmetry imposed	C1	C1
Initial particle images (no.)	496,839	459,520
Final particle images (no.)	95,950	55,670
Map resolution (Å)	3.4	3.5
FSC threshold	0.143	0.143
Map resolution range (Å)	3.0-5.0	3.0-5.0
**Refinement**		
Initial model used (PDB code)	PSI_3-$IsiA_{18}$ (6KIG)	PSI_3-$IsiA_{43}$
Model resolution (Å)	3.4	3.5
FSC threshold	0.5	0.5
Model resolution range (Å)	3.0-5.0	3.0-5.0
Map sharpening *B* factor ($Å^2$)	-99.2	-85.03
Model composition		
Non-hydrogen atoms	235,011	72,843
Protein residues	21,457	6,541
Ligands	1,351	420
B factors ($Å^2$)		
Protein	56.7	40.9
Ligand	61.7	36.5
R.m.s. deviations		
Bond lengths (Å)	0.002	0.006
Bond angles (°)	0.491	0.813
Validation		
MolProbity score	1.61	1.84
Clashscore	12.45	16.56
Poor rotamers (%)	0.42	1.42
Ramachandran plot		
Favored (%)	98.22	98.11
Allowed (%)	1.77	1.89
Disallowed (%)	0.01	0.00

Supplementary Table 2. Calculated FRET rates for EET in PSI_1-$IsiA_{13}$ complex, from the chlorophyll molecules in outer IsiAs to those in inner IsiAs and from the chlorophyll molecules in inner IsiAs to those in the PSI core.

D-protein	D-Chl	A-protein	A-Chl	Distance, R (Å)	Dipole orientation factor, K^2	FRET rate, k_{FRET} (ps^{-1})	Lifetime, τ (ps)
o-1	a519	i-1	a503	27.009	3.928	0.057	17.682
o-2	a504	i-3	a517	22.955	1.848	0.071	14.163
o-3	a508	i-3	a516	19.855	1.789	0.163	6.129
o-3	a519	i-3	a517	21.889	2.688	0.137	7.321
o-4	a510	i-4	a516	26.490	3.701	0.060	16.702
o-5	a519	i-4	a516	19.783	1.144	0.107	9.372
o-5	a504	i-5	a517	23.284	1.747	0.061	16.314
o-6	a519	i-5	a517	20.712	2.061	0.146	6.854
o-6	a508	i-5	a516	21.155	1.189	0.074	13.486
o-7	a510	i-6	a516	27.247	3.670	0.050	19.952
i-1	a519	PsaK	a1103	17.852	1.080	0.186	5.363
i-2	a519	PsaA	a1113	21.000	1.026	0.067	14.958
i-2	a504	PsaA	a1105	24.655	2.363	0.059	17.007
i-2	a508	PsaA	a1108	25.666	2.928	0.057	17.468
i-3	a504	PsaJ	a1303	17.873	2.376	0.407	2.454
i-5	a502	PsaB	a1233	23.508	2.213	0.073	13.646
i-5	a519	PsaX	a1401	23.316	1.484	0.052	19.375
i-5	a504	PsaB	a1233	14.837	1.434	0.751	1.331
i-6	a519	PsaB	a1233	18.001	1.675	0.275	3.635

The lifetime (τ) was defined as $\tau = 1/k_{FRET}$.

Supplementary Table 3. Calculated FRET rates for EET in PSI_3-$IsiA_{43}$ complex, from the chlorophyll molecules in outer IsiAs to those in inner IsiAs and from the chlorophyll molecules in inner IsiAs to those in the PSI core.

D-protein	D-Chl	A-protein	A-Chl	Distance, R (Å)	Dipole orientation factor, K^2	FRET rate, k_{FRET} (ps^{-1})	Lifetime, τ (ps)
o-1	a519	i-1	a503	27.489	3.943	0.051	19.581
o-2	a504	i-3	a517	22.582	1.948	0.082	12.181
o-3	a508	i-3	a516	18.582	1.962	0.266	3.755
o-3	a519	i-3	a517	21.679	2.505	0.135	7.416
o-4	a508	i-4	a506	22.954	1.886	0.072	13.876
o-4	a510	i-4	a516	26.691	3.810	0.059	16.977
o-5	a519	i-4	a516	21.239	0.915	0.056	17.955
o-5	a504	i-5	a517	22.751	1.903	0.077	13.036
o-6	a508	i-5	a516	19.564	2.277	0.227	4.406
o-6	a519	i-5	a517	22.080	2.755	0.133	7.526
o-7	a508	i-6	a506	24.293	2.459	0.067	14.951
o-8	a519	i-6	a503	27.405	3.942	0.052	19.278
o-9	a519	i-7	a516	21.221	1.018	0.062	16.056
o-9	a504	i-8	a517	22.485	1.930	0.083	11.983
o-10	a508	i-8	a516	18.859	1.846	0.229	4.360
o-10	a519	i-8	a517	22.317	2.359	0.107	9.372
o-11	a508	i-9	a506	23.414	2.439	0.083	12.087
o-11	a510	i-9	a516	24.131	2.625	0.074	13.458
o-12	a504	i-10	a517	22.102	1.864	0.089	11.186
o-13	a508	i-10	a516	18.347	1.759	0.258	3.879
o-13	a519	i-10	a517	22.004	2.468	0.122	8.227
o-14	a508	i-11	a506	23.873	2.670	0.081	12.404
o-16	a504	i-13	a517	22.336	2.047	0.092	10.854
o-17	a508	i-13	a516	18.416	1.644	0.236	4.246
o-17	a519	i-13	a517	22.514	2.027	0.087	11.496
o-18	a508	i-14	a506	24.216	2.447	0.068	14.743
o-18	a510	i-14	a516	23.003	1.448	0.055	18.313
o-19	a519	i-14	a503	27.333	3.840	0.051	19.430
o-21	a519	i-16	a517	20.488	1.086	0.082	12.181
o-21	a508	i-16	a506	24.366	2.071	0.055	18.083
o-21	a508	i-16	a516	22.172	1.083	0.051	19.616
o-23	a504	i-18	a517	23.277	1.970	0.069	14.442
o-24	a508	i-18	a516	19.577	1.897	0.188	5.310
o-24	a519	i-18	a517	22.791	2.343	0.093	10.703
o-25	a508	i-1	a506	24.668	2.442	0.061	16.510
o-25	a510	i-1	a516	23.147	1.384	0.050	19.883
i-1	a519	PsaK(1)	a1103	16.949	0.559	0.132	7.592
i-2	a519	PsaA(1)	a1113	20.941	1.226	0.081	12.311
i-2	a508	PsaA(1)	a1108	25.565	3.314	0.066	15.070
i-2	a504	PsaA(1)	a1105	24.380	2.476	0.066	15.171
i-2	a519	PsaA(1)	a1114	14.952	0.123	0.061	16.266
i-3	a504	PsaJ(1)	a1303	17.806	2.304	0.404	2.475
i-3	a519	PsaA(1)	a1105	23.583	1.747	0.057	17.623
i-3	a519	PsaJ(1)	a1302	17.709	0.305	0.055	18.117
i-5	a504	PsaB(1)	a1233	14.780	0.982	0.527	1.900
i-5	a502	PsaB(1)	a1233	23.234	2.778	0.099	10.132
i-7	a519	PsaK(2)	a1103	16.897	0.438	0.105	9.499
i-8	a519	PsaA(2)	a1113	21.176	1.161	0.072	13.897

i-8	a504	PsaA(2)	a1105	24.600	2.515	0.063	15.770
i-8	a508	PsaA(2)	a1108	25.993	3.259	0.059	16.932
i-9	a504	PsaJ(2)	a1303	17.858	2.267	0.391	2.560
i-9	a519	PsaA(2)	a1105	23.725	1.674	0.052	19.055
i-11	a504	PsaB(2)	a1233	14.824	0.936	0.493	2.029
i-11	a502	PsaB(2)	a1233	23.458	2.686	0.090	11.096
i-12	a519	PsaB(2)	a1233	20.313	1.423	0.113	8.832
i-13	a519	PsaK(3)	a1103	17.172	0.530	0.116	8.657
i-14	a519	PsaA(3)	a1113	20.967	1.186	0.078	12.816
i-14	a504	PsaA(3)	a1105	24.072	2.488	0.071	13.993
i-14	a508	PsaA(3)	a1108	25.606	3.279	0.065	15.379
i-14	a519	PsaA(3)	a1114	15.102	0.110	0.052	19.225
i-15	a504	PsaJ(3)	a1303	17.608	2.420	0.454	2.204
i-15	a519	PsaA(3)	a1105	23.249	1.836	0.065	15.391
i-15	a519	PsaJ(3)	a1302	17.422	0.321	0.064	15.603
i-17	a504	PsaB(3)	a1233	15.298	1.222	0.533	1.876
i-17	a502	PsaB(3)	a1233	24.056	2.793	0.081	12.415
i-18	a519	PsaB(3)	a1233	19.919	1.036	0.093	10.786

The lifetime (τ) was defined as $\tau = 1/k_{FRET}$.

Supplementary Table 4. Calculated FRET rates for EET in PSI_3-$IsiA_{43}$ complex with the rotated outer IsiA ring, from the chlorophyll molecules in outer IsiAs to those in inner IsiAs.

D-protein	D-Chl	A-protein	A-Chl	Distance, R (Å)	Dipole orientation factor, K^2	FRET rate, k_{FRET} (ps^{-1})	Lifetime, τ (ps)
o-1	a508	i-1	a516	20.414	1.527	0.118	8.480
o-1	a519	i-1	a517	23.464	2.595	0.087	11.507
o-2	a508	i-2	a506	24.842	2.290	0.054	18.361
o-2	a519	i-2	a517	21.774	0.972	0.051	19.601
o-5	a519	i-4	a517	18.336	0.872	0.128	7.799
o-5	a508	i-4	a506	21.069	1.967	0.126	7.955
o-5	a510	i-4	a516	19.755	1.112	0.105	9.563
o-5	a518	i-4	a517	20.998	1.306	0.085	11.742
o-5	a508	i-4	a516	19.585	0.770	0.076	13.109
o-6	a508	i-5	a506	22.229	1.607	0.074	13.432
o-6	a511	i-5	a506	19.786	0.697	0.057	17.687
o-7	a504	i-6	a517	23.305	2.114	0.074	13.563
o-8	a508	i-6	a516	19.282	1.266	0.138	7.262
o-8	a519	i-6	a517	21.990	1.740	0.086	11.623
o-9	a508	i-7	a506	24.425	2.128	0.056	17.851
o-11	a519	i-8	a516	22.150	1.162	0.055	18.188
o-11	a504	i-9	a517	24.693	2.072	0.051	19.575
o-12	a508	i-9	a516	19.516	1.218	0.123	8.118
o-12	a519	i-9	a517	23.201	1.647	0.059	16.940
o-13	a508	i-10	a506	21.568	1.485	0.082	12.128
o-13	a510	i-10	a516	25.704	3.799	0.074	13.582
o-14	a519	i-10	a516	18.885	0.895	0.110	9.073
o-14	a504	i-11	a517	21.242	2.314	0.141	7.104
o-15	a508	i-11	a516	17.775	1.770	0.314	3.187
o-15	a519	i-11	a517	20.599	2.052	0.150	6.661
o-15	a519	i-11	a501	21.166	1.077	0.067	14.936
o-16	a519	i-12	a517	20.128	1.234	0.104	9.640
o-16	a508	i-12	a506	23.230	2.193	0.078	12.818
o-16	a510	i-12	a516	21.746	1.375	0.073	13.756
o-17	a510	i-15	a516	26.774	3.377	0.051	19.518
o-17	a508	i-15	a506	23.721	2.044	0.064	15.599
o-18	a519	i-13	a516	21.744	1.032	0.055	18.321
o-18	a504	i-14	a517	24.118	2.130	0.060	16.534
o-19	a508	i-14	a516	20.102	1.365	0.116	8.650
o-19	a519	i-14	a517	23.220	2.061	0.073	13.610
o-20	a508	i-15	a506	24.059	2.440	0.070	14.222
o-20	a510	i-15	a516	23.247	1.683	0.060	16.780
o-21	a504	i-16	a517	21.841	1.738	0.090	11.173
o-22	a508	i-16	a516	17.309	1.363	0.283	3.529
o-22	a519	i-16	a517	23.084	2.193	0.081	12.344
o-23	a508	i-17	a506	20.956	2.382	0.157	6.363
o-23	a519	i-17	a517	18.829	1.185	0.149	6.731
o-23	a518	i-17	a517	20.819	1.681	0.115	8.670
o-23	a511	i-17	a516	15.144	0.212	0.098	10.197
o-23	a510	i-17	a516	21.786	1.211	0.063	15.801
o-23	a508	i-17	a516	21.306	0.893	0.053	18.731
o-24	a508	i-18	a506	23.367	1.964	0.067	14.831
i-1	a519	PsaK(1)	a1103	16.916	0.558	0.133	7.510
i-1	a508	PsaK(1)	a1103	22.918	1.348	0.052	19.235

i-2	a519	PsaA(1)	a1113	20.901	1.228	0.082	12.149
i-2	a508	PsaA(1)	a1108	25.101	3.346	0.075	13.375
i-2	a504	PsaA(1)	a1105	24.328	2.496	0.067	14.860
i-2	a519	PsaA(1)	a1114	14.958	0.126	0.063	15.876
i-3	a504	PsaJ(1)	a1303	17.758	2.347	0.418	2.391
i-3	a519	PsaA(1)	a1105	23.470	1.759	0.059	17.007
i-3	a519	PsaJ(1)	a1302	17.667	0.306	0.056	17.784
i-5	a504	PsaB(1)	a1233	14.731	1.010	0.552	1.810
i-5	a502	PsaB(1)	a1233	23.216	2.762	0.099	10.143
i-7	a519	PsaK(2)	a1103	16.857	0.439	0.107	9.345
i-8	a519	PsaA(2)	a1113	21.137	1.170	0.073	13.640
i-8	a504	PsaA(2)	a1105	24.544	2.511	0.064	15.579
i-8	a508	PsaA(2)	a1108	25.542	3.307	0.067	15.021
i-9	a504	PsaJ(2)	a1303	17.781	2.313	0.409	2.444
i-9	a519	PsaA(2)	a1105	23.621	1.688	0.054	18.411
i-11	a504	PsaB(2)	a1233	14.784	0.974	0.521	1.918
i-11	a502	PsaB(2)	a1233	23.441	2.660	0.090	11.161
i-12	a519	PsaB(2)	a1233	20.369	1.474	0.115	8.669
i-13	a519	PsaK(3)	a1103	17.125	0.525	0.116	8.600
i-14	a519	PsaA(3)	a1113	20.924	1.195	0.080	12.569
i-14	a504	PsaA(3)	a1105	24.031	2.498	0.072	13.795
i-14	a508	PsaA(3)	a1108	25.177	3.318	0.073	13.734
i-14	a519	PsaA(3)	a1114	15.108	0.113	0.053	18.830
i-15	a504	PsaJ(3)	a1303	17.546	2.446	0.468	2.135
i-15	a519	PsaA(3)	a1105	23.142	1.843	0.067	14.914
i-15	a519	PsaJ(3)	a1302	17.381	0.330	0.067	14.948
i-17	a504	PsaB(3)	a1233	15.269	1.233	0.544	1.840
i-17	a502	PsaB(3)	a1233	24.033	2.796	0.081	12.328
i-18	a519	PsaB(3)	a1233	19.937	1.041	0.093	10.790

The lifetime (τ) was defined as $\tau = 1/k_{\mathrm{FRET}}$.

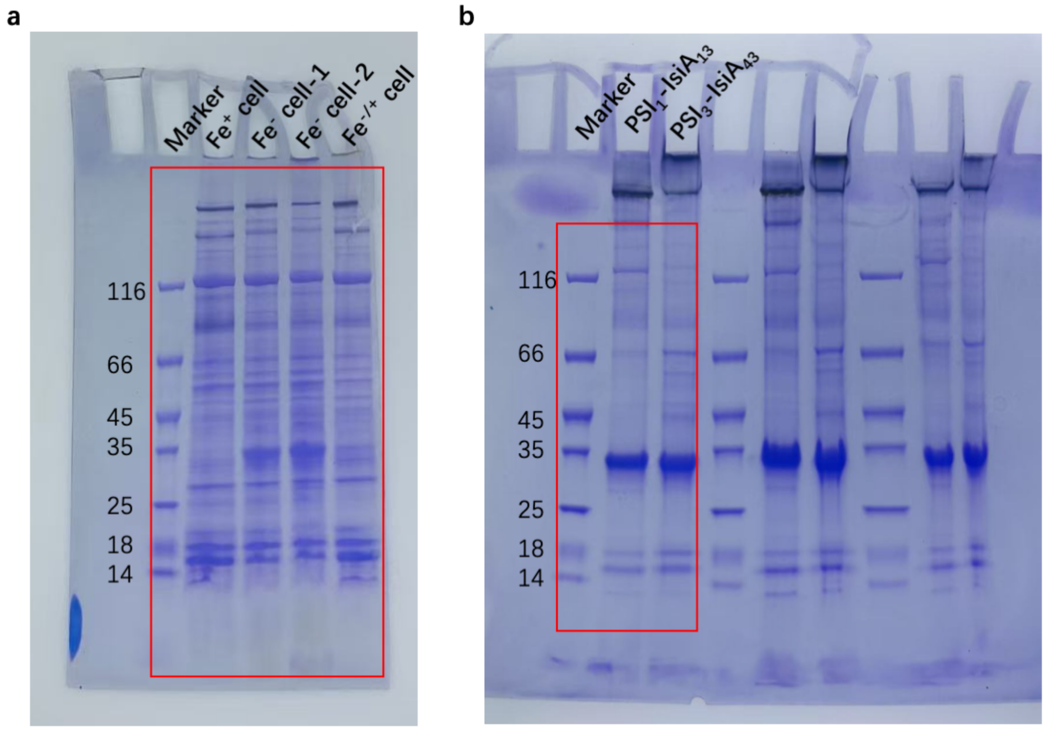

Unedited gel images for Supplementary Figure 1a (a) and Supplementary Figure 3a (b).
